# Partnership and Participation—A Social Network Analysis of the 2017 Global Fund Application Process in the Democratic Republic of the Congo and Uganda

**DOI:** 10.5334/aogh.2961

**Published:** 2020-11-05

**Authors:** Katharine D. Shelley, Carol Kamya, Godefroid Mpanya, Salva Mulongo, Shakilah N. Nagasha, Emily Beylerian, Herbert C. Duber, Bernardo Hernandez, Allison Osterman, David E. Phillips, Jessica C. Shearer, on behalf of the Global Fund Prospective Country Evaluation IHME/PATH consortium collaborators

**Affiliations:** 1 PATH, Seattle, US; 2 Infectious Diseases Research Collaboration (IDRC), Kampala, UG; 3 PATH, Kinshasa, CD; 4 Department of Emergency Medicine, University of Washington, Seattle, US; 5 Department of Health Metrics Sciences, Institute for Health Metrics and Evaluation (IHME), University of Washington, Seattle, US

## Abstract

**Background:**

The Global Fund to Fight AIDS, Tuberculosis and Malaria was founded in 2002 as a public-private partnership between governments, the private sector, civil society, and populations affected by the three diseases. A key principle of the Global Fund is country ownership in accessing funding through “engagement of in-country stakeholders, including key and vulnerable populations, communities, and civil society.” Research documenting whether diverse stakeholders are actually engaged and on how stakeholder engagement affects processes and outcomes of grant applications is limited.

**Objective:**

To examine representation during the 2017 Global Fund application process in the Democratic Republic of the Congo (DRC) and Uganda and the benefits and drawbacks of partnership to the process.

**Methods:**

We developed a mixed-methods social network survey to measure network structure and assess perceptions of how working together in partnership with other individuals/organizations affected perceived effectiveness, efficiency, and country ownership of the application process. Surveys were administered from December 2017–May 2018, initially to a set of central actors, followed by any individuals named during the surveys (up to 10) as collaborators. Network analyses were conducted using R.

**Findings:**

Collaborators spanning many organizations and expertise areas contributed to the 2017 applications (DRC: 152 nodes, 237 ties; Uganda: 118 nodes, 241 ties). Participation from NGOs and civil society representatives was relatively strong, with most of their ties being to different organization types, Uganda (63%), and DRC (67%), highlighting their collaborative efforts across the network. Overall, the perceived benefits of partnership were high, including very strong ratings for effectiveness in both countries. Perceived drawbacks of partnership were minimal; however, less than half of respondents thought partnership helped reduce transaction costs or financial costs, suggesting an inclusive and participatory process may come with short-term efficiency tradeoffs.

**Conclusions:**

Social network analysis can be useful for identifying who is included and excluded from the process, which can support efforts to ensure stronger, more meaningful engagement in future Global Fund application processes.

## Introduction and Background

The Global Fund to Fight AIDS, Tuberculosis and Malaria was founded in 2002 as a public-private partnership between governments, the private sector, civil society, and populations affected by the three diseases. As a financing mechanism, the Global Fund raises and disburses funds to principal recipients (e.g., government agencies, NGOs, or local offices of multilateral organizations) and is among the largest global health initiatives, disbursing nearly US$4 billion per year in over 100 countries toward its mission of accelerating the end of the three epidemics [1].

Four principles guide the work of the Global Fund: country ownership, partnership, performance-based financing, and transparency [2]. The Global Fund encourages country ownership through engaging a variety of in-country stakeholders to harness local perspectives and expertise in decision-making, including governments, bilateral and multilateral donors, the private sector, technical partners, foundations, civil society, representatives from key affected populations, and researchers, among others [3,4]. Partnership has been a distinguishing feature of the Global Fund since its founding. The Global Fund’s concept of partnership supports alignment with national health plans and strategies, as well as harmonization and coordination with other donors and implementers [5]. As defined by Brinkerhoff, “Partnership—a form of intersectoral and multi-actor collaboration—differs from other governance, management, and coordination models by its emphasis on mutuality of shared goals and outcomes across actors or organizations involved [6].” The rise of partnership in global health aligns with the belief that complex issues cannot be solved by a single organization alone, as well as the trend in public administration and the private sector that working in partnership can improve the efficiency, effectiveness, and legitimacy of decision-making processes and resulting solutions. However, despite its popularity as a buzzword, numerous authors have questioned whether global health “partnerships” truly operate as such or are mostly rhetoric [7,8,9,10].

As described in a recent review, there are various technical support partnership models through which the Global Fund operates [11]; here we focus on the partnerships which aim to support the process of applying for and approving new grants. Among the numerous ways in which partnership and country ownership is facilitated through Global Fund policies, the Country Coordinating Mechanism (CCM) is an in-country governance structure created and mandated by the Global Fund to serve as a “mechanism for public-private partnership in the coordination of national disease programs at country level [12].” The CCM is critically important during the Global Fund funding application process for overall coordination of the submission and ensuring transparency, representation, and participation in decision making.

The process for applying for Global Fund funding has evolved substantially throughout the last decade. In 2013, the New Funding Model introduced flexible application windows, simplification through alignment with national strategic plans, predictability of funding for a three-year cycle, and earlier and enhanced Secretariat engagement and support [13]. It also emphasized early engagement with a diversity of stakeholders and partners to identify appropriate interventions that will ensure Global Fund investments “achieve maximum impact [13].” In 2017, the Global Fund introduced further reforms to improve efficiency, including a differentiated application and review process based on country context and allocation level [4].

Country ownership and partnership are critical for promoting harmonization and impactful design of Global Fund investments, but there has been limited evaluation documenting the complexity of stakeholder inclusion, whether and how diverse stakeholders are *meaningfully* engaged, and how this adds value to the efficiency and effectiveness of decision making during the funding request and grant making process [14,15]. The objective of this paper is to examine representation during Global Fund’s 2017 application process and the benefits and drawbacks of engagement and partnership in the Democratic Republic of the Congo (DRC) and Uganda on the outcomes of efficiency, effectiveness, and country ownership.

### Contextual Comparison of the DRC and Uganda

Both the DRC and Uganda are ranked among the bottom 16% of countries on the Human Development Index [16] and each is classified by the Global Fund as a high impact portfolio, meaning the HIV, TB, and malaria burdens are considered “mission critical” to ending the epidemics and the allocation levels are large (above $400 million) (Table 1). During the 2017–2019 allocation period, the DRC (US$527 million) and Uganda (US$465 million) were among the top recipients of Global Fund investments with the third and seventh largest allocations globally [17]. In addition, they were eligible for catalytic investments through matching funds, a complementary funding mechanism for incentivizing eligible countries to align programming with Global Fund strategic priorities [18]. Both countries secured additional matching funds for HIV programming to reduce human rights related barriers to accessing health services, while Uganda also secured matching funds for HIV programming for adolescent girls and young women and the DRC for building resilient and sustainable systems for health (RSSH) and finding missing TB cases [18].

**Table 1:** Contextual comparison of the DRC and Uganda.

Country Characteristics		DRC	Uganda

Population (2018) [16]		84.1 million	42.7 million
Human Development Index Rank (of 189 entries) (2019) [16]		179	159
HIV prevalence (age 15–49) (2018) [19]		0.8 [95% CI 0.6–0.9]	5.7 [95% CI 5.4–6.1]
Tuberculosis incidence rate (per 100,000 people) (2018) [20]		321 [95% CI 208–458]	200 [95% CI 118–304]
Malaria incidence rate (per 1,000 people at risk) (2018) [21]		320	289
**Global Fund Grant Characteristics**		**DRC**	**Uganda**

Portfolio Type^#^		High Impact	High Impact
Challenging Operating Environment (COE)*		Yes	No
Income category^†^		Low Income	Low Income
Funding Requests and type of review^	*TB/HIV*	Tailored	Full
	*Malaria*	Program Continuation	Full
Global Fund Allocation 2017–19 (US$, millions) [17]		$527.1	$465.1
Additional catalytic matching funds (US$, millions) [18]		$16.0	$9.4
Total number of grants signed to-date [22]		26	20
Total investments signed to-date, since 2003 (US$, billions) [22]		$2.00	$1.49

^#^ In 2016, Global Fund’s Differentiation for Impact initiative resulted in three portfolio categories: Focused (<$75 million; lower disease burden); Core ($75–400 million; higher disease burden); High impact (>$400 million; mission critical disease burden) [23]. * The COE policy was approved by the Global Fund Board in April 2016 to provide guidance on Global Fund engagement in COE contexts through the principles of flexibility, partnerships, and innovation [24]. ^†^ Global Fund’s income level eligibility is based on the World Bank (Atlas Method) Income Classifications, using the latest three-year average of gross national income per capita data to determine income classification thresholds in 2016 [25]. ^ A differentiated funding request model was introduced in 2017 to further streamline the application process [4].

## Methodology

### Global Fund Prospective Country Evaluation

This study is embedded in the Global Fund Prospective Country Evaluation (PCE). The Global Fund’s Technical Evaluation Reference Group (TERG) commissioned the PCE in 2017 to support the independent assessment of implementation and impact of the Global Fund Strategy 2017–2022 [2,26]. The PCE is a three-year prospective evaluation in eight countries—Cambodia, DRC, Guatemala, Mozambique, Myanmar, Senegal, Sudan, and Uganda—to inform Global Fund business model processes by examining what is working, what is not working, and why [27]. The evaluation platform utilizes a mixed-methods approach, covering the full results chain from inputs to impact, and following Global Fund processes prospectively through the grant cycle, including application, approval, preparation, and implementation.

### General methods

Through the PCE platform, this comparative, mixed-methods social network analysis draws from data collected on two cases: the Global Fund application process in the DRC and Uganda. In 2017, the Global Fund introduced a new application approach to enable a more streamlined and efficient process, allowing countries to opt for less burdensome grant continuation processes, as well as the existing full application (Supplemental File 1: Application types) [4]. The DRC submitted funding requests using the tailored review (HIV/TB) and program continuation review (malaria) approaches; whereas, Uganda submitted HIV/TB and malaria funding requests both via full review.

Global Fund application processes are important cases for case study research and policy analysis because of the magnitude of resources tied to them and their instrumentality as key decision-making processes in low-income countries. Per previous partnership analyses, a “case” can be defined as a process with a specific resulting outcome, a feature that also enables stronger cross-country comparison [7,14]. We considered the Global Fund application process as a case for each country: a time-bound process with a specified outcome (submission of the funding requests) reviewed by a panel of experts (Global Fund’s Technical Review Panel) who either recommend proceeding to grant making or send the application back for further iteration to address technical or programmatic weaknesses before resubmission to the Technical Review Panel.

### Sampling Methods and Survey Administration

Social network surveys were administered between December 2017 and May 2018 in Uganda and the DRC by a mixed-methods evaluation team at the Infectious Diseases Research Collaboration (Uganda) and PATH (DRC), the respective PCE country evaluation partners. We defined network members as any actors involved in the Global Fund grant application process. An initial list (i.e., network roster) of potential survey respondents was developed through ongoing document review and meeting observation, which served to identify a subset of individuals likely to be central to the application network, for example including stakeholders from the CCM, national health programs and other government agencies, technical partners, bilateral funders, and civil society. These stakeholders were contacted for an interview about the funding request process. Following the interview, a network survey was administered in person via paper or via an online link to an electronic survey programmed in SurveyGizmo’s data collection platform [28]. In Uganda, data were collected via two rounds: an initial round of surveys was conducted with 14 central actors, and any collaborators named were subsequently contacted and the link to the network survey was shared or an in-person meeting was scheduled (akin to “snowball” or respondent-driven sampling) [29]. In DRC, evaluators compiled a roster and conducted surveys during key informant interviews. In both countries, to increase response rates, stakeholders were followed up with phone call and email reminders. In addition, at the PCE dissemination meetings in April 2018, evaluation teams again shared the network survey link and printed tools as a final attempt to reach any additional respondents involved in the process.

### Survey Instrument

We adapted a structured social network survey from a similar survey conducted by Kamya and colleagues, which drew from an existing conceptual framework of how development partnerships add value to decision-making processes through efficiency, effectiveness, and country ownership [7]. The team refined the survey following document review of Global Fund application guidance, stakeholder interviews, and observation of application-related meetings. The survey was pre-tested among the study team and adjustments were incorporated to improve question clarity and design (Supplemental File 2: Survey instruments).

During the survey, the study team first asked respondents to identify which Global Fund applications they worked on (HIV, TB, malaria, or any combination), and specifically which aspects of the application they supported. Respondents were then asked to provide the names and organizations of up to 10 individuals they personally collaborated with on the Global Fund grant application, which funding request(s) they collaborated on, and to rate their level of professional trust (a strong predictor of network performance [30]) with those individuals using a 4-point scale: 1–Poor relationship (little trust); 2–Fair relationship (some trust); 3–Good relationship (trust); 4–Excellent relationship (high trust), where trust was defined as trusting “the individual or organization to keep their word, do a good job, and respond to your professional needs or your organization’s needs.” The survey closed by asking respondents to indicate whether a benefit or drawback “occurred” or “did not occur” from working in partnership with other individuals or organizations during the 2017 Global Fund application cycle. The survey included 14 potential benefits and 6 potential drawbacks, as adapted from Provan and Milward [31] and Kamya and colleagues [7], to assess collaborator perceptions of how working together in partnership affected effectiveness, efficiency, and country ownership of the process (see Table 4 for benefits and drawbacks organized by domain). For example, “Increased quality and technical soundness of the approved grants” was an indicator of effectiveness; “Reduced transaction costs (i.e., more streamlined grant application process)” was an indicator of efficiency; and “Increased inclusiveness of key stakeholders in the process” was an indicator of country ownership (Supplemental File 2: Survey instruments).

### Analysis

We used existing mathematical algorithms to measure common network metrics, including nodes, ties, density, degree centralization, and betweenness centrality (defined below in Table 3). Each node in the network represents one individual from the original roster or named in the survey. There is a tie between nodes when a survey respondent has reported collaboration. A node’s degree centrality is the number of ties that node has in the network.

To limit the time required to conduct the survey, we capped the number of reported collaborative ties at 10 and provided respondents with a definition of collaboration: “by working together you may have exchanged information to support the funding request, cooperated on writing sections of the funding request, collaborated on setting performance targets and developing action plans, or responded to reviewer comments, etc.” Though not all collaborators named in the survey responded with their own accounts of collaboration, all ties are assumed to be mutual (i.e., undirected, reciprocal) due to the nature of collaboration within partnerships, which are characterized by mutual decision making and reciprocal accountability, distinct from contractual relationships characterized by hierarchical decision making and unilateral accountability [7]. Within the Global Fund application network, we assume information exchange was likely both sent and received between collaborators and thus assumed all ties to be undirected as is typical of partnership alliances [32].

Networks were visualized according to several subgroup attributes available for all nodes, including funding request type, organizational affiliation, gender, and national versus sub-national representation (DRC only). All analyses were conducted using the statnet suite of network analysis packages in the R statistical programming language and the associated statnetWeb R Shiny application [33,34].

Through the overall Prospective Country Evaluation, evidence from document review, meeting observation, and interviews on the funding request process (including transparency, inclusiveness, country ownership, and efficiency) were routinely entered into an analysis matrix, an Excel-based framework for organizing data by thematic area and stakeholder group [35]. To support interpretation, this qualitative data (as summarized in our project reports [36,37]) was triangulated with the data on network characteristics and structure, as well as network member perceptions of efficiency, effectiveness, country ownership, and levels of professional trust among collaborators.

### Research ethics

The DRC protocol was approved by the University of Kinshasa School of Public Health Ethics Committee (#ESP/CE/074/2017), and the Uganda protocol was approved by the Makerere School of Medicine Research Ethics Committee (#REC REF 2017-146) and the Uganda National Council for Science and Technology (#SS 4472). This was determined to be non-human subjects research by the University of Washington’s IRB (#STUDY00003643). The purpose of the survey was explained, including confidentiality measures, and participants were asked for their consent to participate.

## Results

### Context

In both the DRC and Uganda, the 2017 funding requests were submitted during the first application window (March 2017) and approved or validated by the Technical Review Panel to move forward to the grant making stage. There was broad consensus in both countries that the 2017 application process was a success in that it led to grant signature within the planned time frame, allowing countries to avoid a funding gap by remaining on track for initial grant disbursement in early 2018. Stakeholders attributed the faster grant processing to application process changes introduced by the Global Fund, better levels of preparation by country stakeholders, more coordination, and increased support and engagement from the Global Fund Country Teams from the beginning of the application cycle [36,37].

### Network Characteristics

The 2017 Global Fund application required a large network of actors spanning many different types of organizations and expertise. The characteristics of identified actors in the DRC and Uganda application networks are compared in Table 2. In the DRC, 40 surveys were completed from a roster of 67 stakeholders approached to participate (60% response rate) from December 2017 to May 2018 in Kinshasa, Tshopo, and Maniema Provinces, generating a total network of 152 nodes (individuals) with 237 ties (collaboration relationships). In Uganda, 30 surveys were completed from January to May 2018 in Kampala through two rounds of data collection: 14 respondents were initially surveyed, which generated 51 additional unique collaborator names—all were approached, of which 16 completed the survey (46% response rate: 30/65), generating a total network of 118 nodes with 241 ties. Table 3 provides a summary comparison of key network metrics and their definitions. The average survey respondent in the DRC reported 7 collaborators; whereas, the average survey respondent in Uganda reported 11 collaborators; however, the average number of ties reduced to 3 and 4 in the DRC and Uganda, respectively, when considering all identified nodes. Levels of trust between collaborators were generally high in both countries, with a mean of 3.7 in Uganda and 3.4 in the DRC.

**Table 2:** Characteristics of identified actors in DRC and Uganda by funding request, gender, and organizational affiliation.

	DRC			Uganda		

Funding Request	Respondent	Named in survey	Total N (% of total)	Respondent	Named in survey	Total N (% of total)

TB/HIV request only	21	54	75 (49.3%)	13	39	52 (44.1%)
Malaria request only	8	43	51 (33.6%)	6	24	30 (25.4%)
Both	9	15	24 (15.8%)	11	25	36 (30.5%)
Unknown	2	0	2 (1.3%)	0	0	0 (0.0%)
**Gender**						

Male	32	82	114 (75.0%)	17	47	64 (54.2%)
Female	8	30	38 (25.0%)	13	41	54 (45.8%)
**Organization Type**						

NGO/civil society	7	29	36 (23.7%)	5	13	18 (15.3%)
Technical partners	5	25	30 (19.7%)	4	25	29 (24.6%)
Principal Recipient: Gov^#^	10	14	24 (15.8%)	13	19	32 (27.1%)
Principal Recipient: NGO	4	12	16 (10.5%)	3	5	8 (6.8%)
Sub Recipient: NGO	5	15	20 (13.1%)	–	–	–
Government (other)*	6	11	17 (11.2%)	1	10	11 (9.3%)
Consultant	–	–	–	0	8	8 (6.8%)
CCM	1	6	7 (4.6%)	3	3	6 (5.1%)
Local Fund Agent	1	0	1 (0.7%)	1	2	3 (2.5%)
Global Fund	–	–	–	0	3	3 (2.5%)
Unknown	1	0	1 (0.7%)	–	–	–
Totals	40 (26.3%)	112 (73.7%)	152 (100%)	30 (25.4%)	88 (74.6%)	118 (100%)

^#^ Gov = Government; In DRC, the Ministry of Health serves as the Principal Recipient for the public sector; whereas, in Uganda the Ministry of Finance is the Principal Recipient for the public sector (executing entity) and the Ministry of Health serves as the implementing entity (for the purpose of this table they are grouped). * Includes other government agencies, departments, or ministries (e.g., Ministry of Gender, Ministry of Education, Ministry of Justice, Armed Forces, Essential Medicines/Supply Chain, Information Systems, National Health Accounts, excluding Ministry of Health which is captured under Principal Recipient: Gov).

**Table 3:** Comparison of network attribute definitions, values, and interpretation in the DRC and Uganda.

Attribute	Definition	DRC	UGA	Comparison between DRC and Uganda

Node	An individual actor. The number of nodes denotes the network size or the total number of individuals contributing to the application process.	152	118	The network of individuals contributing to the application process in the DRC and Uganda is quite large. In both countries, there were slightly more identified nodes in the TB/HIV network (DRC: 99; Uganda: 64) than the malaria network (DRC: 75; Uganda: 49), noting that some individuals worked across both funding requests.
Tie	Link between two nodes, indicating collaboration between two individual actors working on the application process.	237	241	We assume all relationship ties were *undirected* (e.g., mutual; collaborative) during the Global Fund application process. In the DRC, more ties were identified in the TB/HIV network (169) than the malaria network (92). Similarly, in Uganda, more ties were identified in the TB/HIV network (176) than the malaria network (108). Ties where the collaborator worked on both funding requests are counted in both categories.
Average degree centrality	Average number of ties per node, meaning the average number of individuals each actor collaborated with.	3	4	The average node in the DRC had 3 ties, meaning the average individual actor collaborated with 3 individuals. Among individual nodes that responded to the survey, on average each reported 7 ties. Averages were slightly higher in Uganda: 4 ties per node, and 11 ties per respondent node. This suggests the overall density of ties would increase with a higher survey response rate.
Isolate	Unconnected node: an individual actor named in the survey with no collaborative ties to other individual actors.	3	4	In addition to listing up to 10 individuals with whom the survey respondent collaborated, respondents were asked who was “most influential” in the application process. In DRC (n = 3) and Uganda (n = 4), this resulted in isolates; however, these may not be “true” isolates given the survey response rate.
Density	Number of existing ties divided by the number of possible ties.	0.02	0.04	The relatively low density (meaning 2 to 4% of potential ties exist) should be interpreted with caution given the moderate survey response rate.
Degree centralization	Extent to which the network is dominated by one or a few focal actors.	0.09	0.16	The medium-to-low degree centralization score for the DRC (0.09) and Uganda (0.16) networks are indicative of a decentralized network with multiple collaboration hubs across funding requests, which are important for information exchange and settings requiring multiple focal actors across intersecting groups.
Betweenness centrality	Extent to which a node is located on the shortest paths between other actors.	See Figure 2 in Supplemental File 3		Actors with high betweenness centrality scores serve as bridges: they are in a structural position to control the flow of information and to most efficiently transfer information to the greatest number of other actors in the network.
Mean reported trust	Average trust score in the network.	3.4	3.7	Survey respondents were asked to rate levels of perceived trust (on a scale of 1 to 4) with each of the collaborators they named. The high levels of trust between individuals is indicative of strong collaborative relationships in the DRC and Uganda.

### Network Structures by Node Attribute: Funding Request, Gender, and Organizational Affiliation

**Figure 1:**
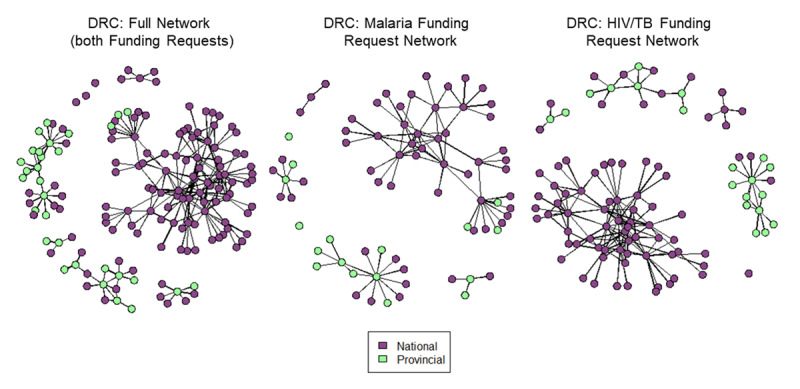
DRC’s 2017 Global Fund application network with nodes represented by national vs. provincial-level stakeholders for the full network and disaggregated by malaria and TB/HIV funding requests.

The DRC’s overall network structure is characterized by a larger, central core and several peripheral collaboration hubs. The peripheral hubs tend to have more representation from provincial-level stakeholders (Figure 1), while the central hub is nearly entirely composed of national-level stakeholders. In the 2017 application cycle, some provincial stakeholders were invited to participate in the national country dialogue, and additional country dialogues were hosted in a few select provinces. Subnational participation was not captured by the network survey in Uganda.

#### Funding Request Type

**Figure 2:**
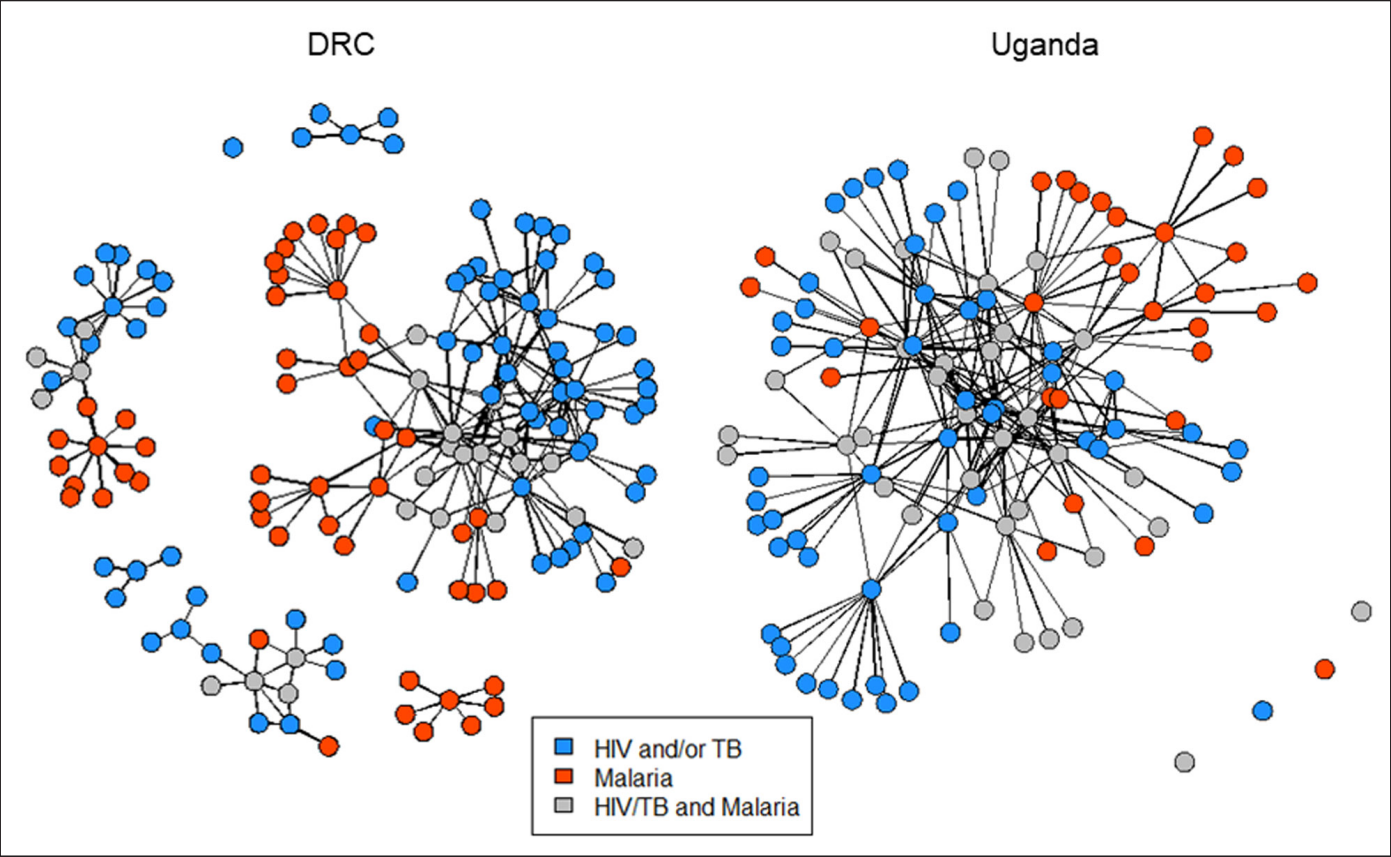
Plots of Uganda and DRC’s 2017 Global Fund application networks with nodes represented by funding request type. *Note*: A few isolates, or unconnected nodes without ties to others, were identified in each network from survey respondents listing names for the “most influential” member of the network but who were not otherwise named through the listing of individual collaborators—this might be indicative of influential leaders crucial to decision making but not involved in the collaborative work of developing the funding requests.

Global Fund application network structures were examined by funding request type, with nodes color coded for TB/HIV (blue), malaria (red), or both funding requests (grey) (Figure 2). In Uganda, nearly one third of the network (31%) collaborated on both funding requests; whereas, in the DRC only 16% collaborated on both, which is visually apparent in the network plots where more of Uganda’s central core includes light grey nodes. In both countries, over two thirds of the network collaborated on the TB/HIV funding request (blue and grey nodes), including 65% of the DRC’s network and 75% of Uganda’s network; whereas, closer to half collaborated on the malaria funding request (red and grey nodes) in the DRC (49%) and Uganda (56%). Stakeholder interviews indicated that collaboration between the DRC’s HIV and TB national programs to develop the joint application was perceived to be more concerted and coordinated than in past application cycles [36].

#### Gender

**Figure 3:**
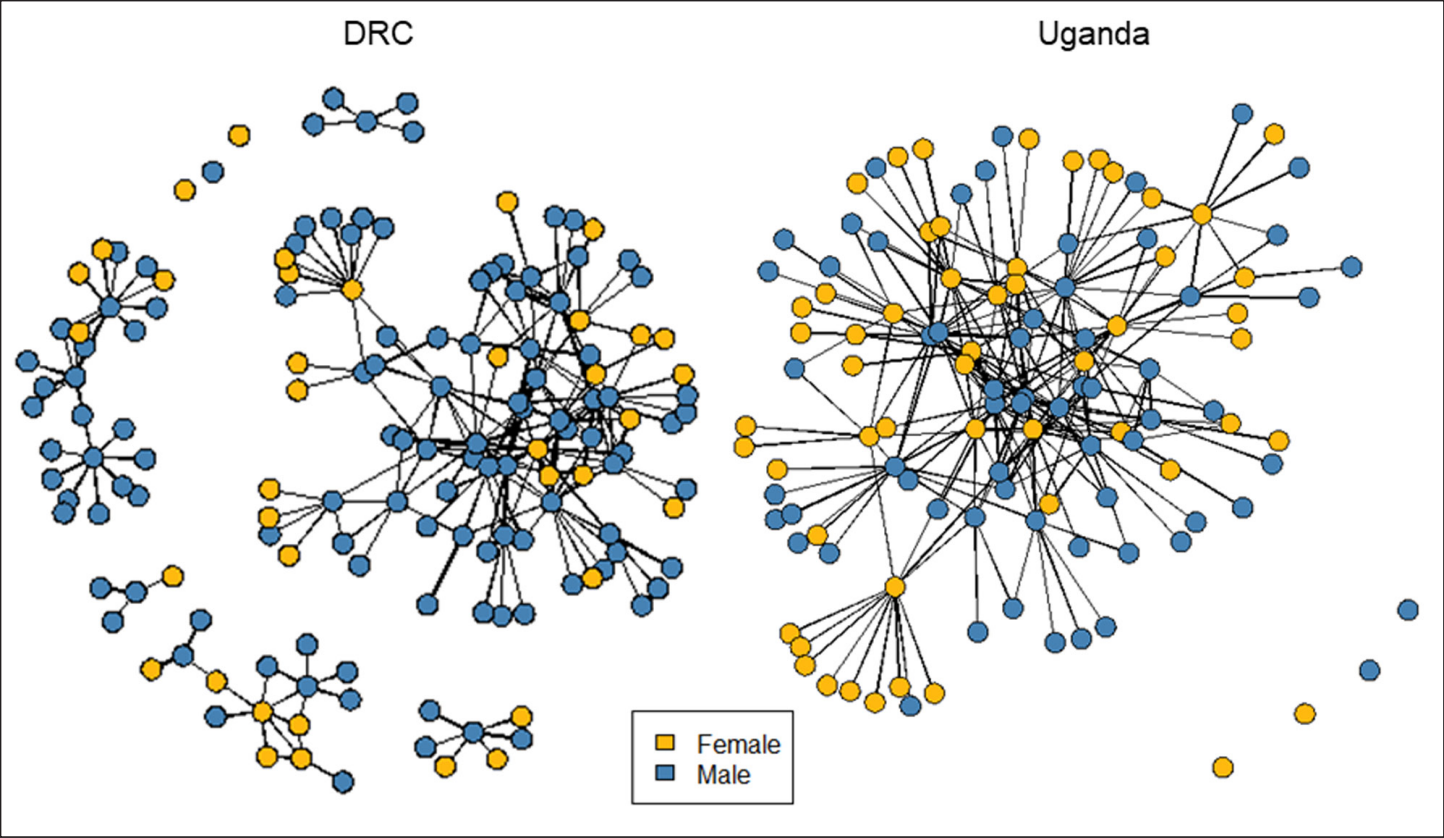
Plots of Uganda and the DRC’s 2017 Global Fund application networks with nodes represented by gender.

When the full networks are plotted by gender (Figure 3), the structure suggests that in the DRC there were more males (75%, blue nodes) and males held a more dominant and influential position at the network’s center. Among the 38 females identified in the DRC network, 42% were part of smaller, peripheral networks unconnected to the larger, core network. In contrast, in Uganda the network was notably more gender balanced (54% male, blue nodes), with males appearing to hold a slightly more influential position in the central core of the network. In examining the malaria and TB/HIV requests separately, in Uganda, females comprised a greater proportion of the TB/HIV application network (48%) than the malaria network (39%); whereas, there was no difference in female representation comparing the DRC’s TB/HIV network (26%) and malaria network (25%) (Supplemental File 3, Figure 1).

#### Organizational Affiliation

**Figure 4:**
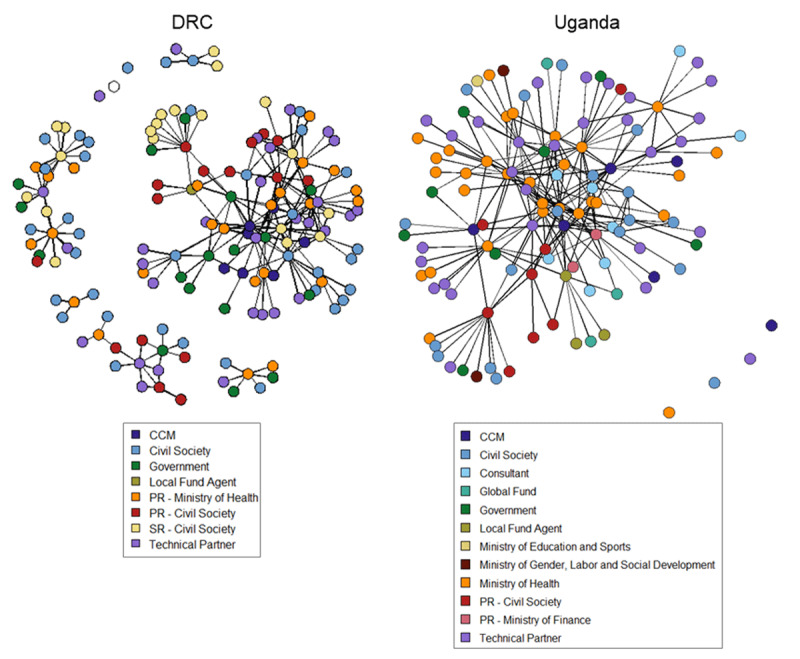
Plots of Uganda and DRC’s 2017 Global Fund application networks with nodes represented by organizational affiliation.

In both the DRC and Uganda, roughly 70% of the network was composed of individuals representing civil society, technical partners, or Principal Recipients (public sector or non-governmental organization) (Table 2) and there was balanced involvement of actors representing a variety of organizational affiliations (Figure 4; Supplemental File 3, Figure 2). Within the DRC’s malaria network, the Ministry of Health (orange), civil society principal recipient (red), other government actors (dark green), and CCM (dark blue) were the most central and strategically positioned collaborators, as measured by their degree centrality; whereas, within the TB/HIV network, civil society (medium blue), the Ministry of Health (orange), the civil society sub recipient (yellow), technical partners (lavender), and CCM held the most central positions based on degree centrality (Supplemental File 3, Figure 3). In Uganda’s full network, the Ministry of Health (orange), CCM (dark blue), and technical partners (lavender) had the highest degree centrality (Figure 4; Supplemental File 3, Figure 2). The Ministry of Health, CCM, and Ministry of Finance (a principal recipient; light red) had the highest degree centrality in Uganda’s malaria network, which was smaller and less dense compared to the TB/HIV network where the Ministry of Health, technical partners, CCM, and civil society principal recipient (red) were most central (Supplemental File 3, Figure 3). Consultants played a more central role in Uganda’s TB/HIV request than malaria request, as measured by average degree centrality (Supplemental File 3, Figure 3). The increased role of local consultants (n = 7) versus international consultants (n = 1) during Uganda’s 2017 application phase was emphasized in stakeholder interviews as an important factor for success, compared to the reliance on international consultants during prior application cycles [28]. The network plots suggest a very central position of the consultants (light blue), consistent with their important contribution to the development and writing of the application and the need to coordinate across numerous types of stakeholder groups providing inputs.

NGOs and civil society (including civil society PRs and SRs) were well represented in the application networks of both the DRC (47%) and Uganda (22%) (Table 2, Figure 5). These organizations were also well connected and integrated within the network: In Uganda, 40 of 64 civil society ties (63%) were with other organization types, while in the DRC 100 of 150 civil society ties (67%) were with other organization types. In both countries, the civil society organizations were better connected (average degree centrality) and more influential (as connectors/brokers as measured by average betweenness scores) in the TB/HIV network than the malaria network (Supplemental File 3, Figure 3). In Uganda, civil society (medium blue), including representatives of key and vulnerable populations, appeared clustered together and at the peripheral edges of the network, which could be a result of these actors working within one umbrella organization (Civil Society Network) for representation and coordination purposes.

Participation by other government ministries, including the Ministry of Gender, Labor and Social Development (maroon) and Ministry of Education and Sport (light yellow), is also notable in Uganda given the increased emphasis on HIV programming for adolescent girls and young women (via the catalytic matching funds for HIV); however, participation was limited (n = 3), peripheral, and did not include representatives with decision-making authority.

### Perceived Benefits and Drawbacks of Partnership

Survey respondents were asked about perceived benefits and drawbacks of partnership that occurred due to working together with other individuals and organizations in supporting the 2017 Global Fund application process. The percent agreement with each of the potential benefit and drawback statements, organized according to effectiveness, efficiency, and country ownership domains, is shown in Table 4. Overall, respondents in Uganda rated partnership benefits relatively higher than respondents in DRC. Perceived drawbacks of partnership were minimal in both countries.

**Table 4:** Perceived benefits and drawbacks of partnership in DRC and Uganda.

Perceived benefits of partnership	Agreed “occurred”	

Effectiveness	DRC	Uganda

Increased quality and technical soundness of the approved grants	28 (78%)	27 (100%)
Better able to execute activities	28 (78%)	25 (93%)
Better able to respond to challenges and bottlenecks that arose during process	28 (78%)	25 (93%)
Better able to identify the need for, and to acquire, additional technical support	30 (83%)	23 (85%)
*Mean (effectiveness benefits)*	*79%*	*93%*
**Efficiency**	**DRC**	**Uganda**

More timely execution of planned activities	21 (58%)	25 (93%)
Leveraged each organization’s comparative advantages	16 (44%)	23 (85%)
Reduced transaction costs (i.e., more streamlined grant application process)	13 (36%)	13 (48%)
Reduction in financial cost of process	12 (33%)	5 (19%)
*Mean (efficiency benefits)*	*43%*	*61%*
**Country ownership**	**DRC**	**Uganda**

Approved grants that are more responsive to country needs	15 (42%)	25 (93%)
Increased inclusiveness of key stakeholders in the process	27 (75%)	23 (85%)
Increased fairness of decisions made	27 (75%)	23 (85%)
Increased legitimacy of decisions made	28 (78%)	22 (81%)
Increased accountability among partners	26 (72%)	21 (78%)
Increased transparency among partners	26 (72%)	21 (78%)
*Mean (country ownership benefits)*	*69%*	*83%*
**Perceived drawbacks of partnership**	**Agreed “occurred”**	

**Effectiveness**	**DRC**	**Uganda**

Created competition and conflict among member organizations	11 (31%)	8 (30%)
Strained relations within my organization	4 (11%)	4 (15%)
*Mean (effectiveness drawbacks)*	*21%*	*23%*
**Efficiency**	**DRC**	**Uganda**

Forced to make decisions in a way which was not natural/typical for our organization	7 (19%)	7 (27%)
Loss of control/autonomy over decisions	2 (6%)	4 (15%)
Unnecessary management burden on my organization	7 (19%)	2 (8%)
*Mean (efficiency drawbacks)*	*15%*	*17%*
**Country ownership**	**DRC**	**Uganda**

Not enough credit given to my organization	3 (8%)	4 (15%)
*Total (country ownership drawbacks)*	*8%*	*15%*

#### Effectiveness

Across four items, partnership effectiveness benefits were rated higher by respondents in Uganda (93%) than the DRC (79%) on average. In Uganda, 100% of respondents agreed that partnership had improved the quality and technical soundness of the approved Global Fund grants; whereas, in the DRC most respondents (79%) agreed with the statement. This triangulates with evidence that Uganda’s grant applications received minimal comments from the Technical Review Panel and that grant applications were reviewed and approved on time in both countries. Most respondents in the DRC (83%) and Uganda (85%) were also better able to identify the need for, and to acquire, additional technical support. Nearly one third of respondents in the DRC (31%) and Uganda (30%) reported a drawback of partnership was that it created competition and conflict among member organizations.

#### Efficiency

There was general agreement that partnership supported the efficiency of the grant application process in terms of timely execution of planned activities in Uganda (93%), but this benefit was much less frequently perceived to have occurred in the DRC (58%). Most respondents in Uganda agreed that partnership helped to leverage an organization’s competitive advantages (85%) during the grant application, but fewer than half of the DRC respondents (44%) agreed. However, fewer than half responded that partnership helped to reduce transaction costs (Uganda: 48%; DRC: 36%) or helped reduce the financial cost of the process (Uganda: 19%; DRC: 33%). Overall, the perceived drawbacks of the partnership on efficiency were minimal: some respondents in the DRC (19%) and Uganda (29%) thought it resulted in making decisions in an unnatural or atypical way, and few respondents reported strained relations, loss of control/decision-making autonomy, or lack of credit as drawbacks that occurred.

#### Country Ownership

While nearly all respondents in Uganda (93%) perceived partnership to have contributed to approved grants that were more responsive to country needs, only 42% of respondents in the DRC responded similarly. Over three quarters of respondents in both countries perceived partnership to have resulted in increased inclusiveness of stakeholders, increased accountability and transparency among partners, and increased fairness and legitimacy of decisions.

## Discussion

Social network analysis has increasingly been applied to understanding complex health systems and decision-making structures, such as stakeholder roles and relationship composition within networks, including who is included or excluded, which organizations/actors are most influential, and how relationships form network structures that can influence processes and outcomes [14,38,39,40,41]. Application of social network analysis to the 2017 Global Fund grant application process in the DRC and Uganda indicates both were highly inclusive processes in terms of stakeholder participation, characterized by strong representation from the government, technical partners, and civil society organizations, and high levels of trust among collaborators. The implications of the findings for future Global Fund application cycles are discussed.

The Global Fund grant application process is a resource-intensive undertaking, requiring about nine months of engagement from a variety of stakeholders. Our analyses indicate well over one hundred stakeholders were involved in the development of the funding requests in the DRC and Uganda, representing a diverse set of organizations and suggesting the process was highly inclusive (in line with the Global Fund principle of “engagement of in-country stakeholders, including key and vulnerable populations, communities, and civil society” in accessing funding [4]). That the civil society representatives in both Uganda and the DRC were engaged and had strong collaborative linkages to other organizational types is an encouraging finding, given that a network with ties both within and across subgroups helps promote information transfer across areas of expertise. An analysis by Shearer and colleagues in Burkina Faso demonstrated that a greater diversity of participants within a network is associated with more exposure to new ideas and evidence, which in turn can lead to more innovative policy decisions [14].

Given the Global Fund’s strategic objective to “promote and protect human rights and gender equality [2],” it is essential that civil society representatives for key affected populations are included in the funding request development process in *meaningful* ways. Interview data in Uganda highlighted strong participation of key and vulnerable populations and gender and human rights constituencies in the 2017 application phase compared to the previous funding cycle [37]. Similarly, many of the stakeholders in the DRC, including those most closely involved in preparing the funding request, indicated the process was highly inclusive, participatory, and that all the major stakeholder groups were represented in the country dialogue. However, some civil society members interviewed did not think the process had adequate and meaningful representation, in that their participation was not taken seriously and instead served the purpose of meeting a Global Fund requirement [36]. Given that government, CCM, and technical partner collaborators were more central and therefore more influential within the network, we hypothesize this may have contributed to some civil society actors perceiving their participation as less influential, despite overall strong inclusiveness.

Gendered power relations within health policy making processes can affect participation within governance structures, including who is able to engage, their terms of engagement and how accountability is negotiated between actors [42]. A checklist tool for integrating gender into the processes and mechanism of the Global Fund application can help promote inclusion of gender dimensions in national plans and areas where Global Fund invests [43], but does not include, an assessment of *who* is involved in the application development, including for priority setting and decision making. Our data indicate only 25% of the application network in the DRC was female; whereas, stronger balance (46% female) was evident in Uganda’s network—a finding which may reflect prevailing norms in government leadership, with the share of parliament seats held by women higher in Uganda (34.8%) relative to the DRC (8.2%) [11]. Through their coordination role, the CCM could help in fostering more gender balance within the network supporting application development in future grant cycles.

The Global Fund’s ability to reform is recognized as a unique strength of the organization [44], and recent reforms to differentiate the application process deserve further exploration to ensure they are delivering on intended goals of streamlining and efficiency. Relative to Uganda (>85%), many fewer respondents in the DRC perceived partnership to have contributed to approved grants more responsive to country needs (42%), to have supported more timely execution of planned activities (58%), or to have leveraged an organization’s competitive advantages (44%). We hypothesize that some of this difference can be attributed to the program continuation and tailored review application approaches undertaken in the DRC, which were designed for streamlining through maintaining the strategies of the prior 2015–2017 grants, with potentially less opportunity for integrating new inputs and innovations but a similarly large number of stakeholders participating in the country dialogue. The relatively larger network size in DRC (152) compared to Uganda (118) might also help to explain some differences in perceived efficiency and responsiveness. Notably, less than half of respondents felt working in partnership helped to reduce transaction costs (Uganda: 48%; DRC: 36%), and fewer thought it helped to reduce the financial cost of the process (Uganda: 19%; DRC: 33%). These lower efficiency ratings align with interview data suggesting increased transaction costs associated with a highly inclusive and participatory application process [36,37].

Considering the potential downsides of highly participatory processes is also important—despite being difficult to quantify, these issues are felt in terms of the duration of the process and associated opportunity costs. Our evidence suggests alternative models for “right-sizing” participation and inclusion to ensure meaningful engagement is balanced with efficiency is worth further consideration by the Global Fund. Similarly, recent research on the 2017 funding request process in Malawi, Tanzania, and Zimbabwe questions the relevance and effectiveness of Global Fund’s processes for multisectoral inclusion and deliberative engagement and whether a lengthy country dialogue process is necessary to determine spending priorities. In the context of increasingly commoditized grants where much of the allocation is predetermined (and thus the need for further deliberation and prioritization across treatment, prevention, and systems strengthening is reduced), such processes may be functioning “as processes for processes’ sake alone [15].” More research is necessary to understand whether and how degree of inclusiveness is resulting in stronger prioritization, greater responsiveness to country needs, and higher quality, more technically-sound funding requests.

Global Fund’s mandate is to end the three diseases, but increasingly, investments will need to support the Universal Health Coverage [45] and Sustainable Development Goals agenda [46], and funding requests will require a multisectoral approach that further engages experts beyond health. Our network analyses indicate Uganda has made strides in this direction to support the design and implementation of investments for adolescent girls and young women, but the peripheral involvement of the Ministry of Education and Ministry of Gender was insufficient. More active and central engagement will be required to promote coordination across ministries to ensure alignment of Global Fund investments with the mandates and strategic plans across sectors and to secure cross-sectoral buy-in not just for the design but also for the implementation of key activities. A core function of the CCM is to lead and govern the development of the funding request through multi-stakeholder engagement [4]. The network analysis highlights the central role of the CCM in coordinating and developing the funding request, and they are well positioned to play a stronger role in fostering multisectoral collaboration not just during the application phase, but also during grant implementation. Both Uganda and the DRC were among 18 countries that participated in the Global Fund “CCM Evolution’’ pilot project aimed at strengthening CCM oversight and functioning [47], which could prove useful in improving multisectoral engagement during the 2020 application process, as well as in facilitating a strong and coordinated effort to utilize Global Fund flexibilities in rapidly responding to the COVID-19 pandemic.

### Strengths and Limitations

The study was guided by a theoretical framework on partnership, adapted from an evaluation of a large global health initiative [7,9]. Utilizing network analysis is an overall strength of the approach, given the paucity of applied quantitative or mixed methods designs to the evaluation of multisectoral collaboration [48]. Disaggregation of social network visuals by key characteristics of nodes can support further analyses, which is an important contribution to examining assumptions about participation and inclusiveness in partnership driven processes.

The survey was undertaken shortly following the grant signing in each country to limit participant recall bias. Potential differences in respondent bias between countries may help explain some of the differences in responses to questions about perceived effectiveness, efficiency, and country ownership of the process. Based on our observation of the funding request process and review of meeting attendance records, we are fairly confident that the survey captured the majority of network members; however, the low survey response rate is a limitation, which reduces our confidence in the true structure of the network given that a higher response rate would yield a more accurate reflection of the network’s density. We assumed all collaborative ties to be undirected (mutual); however, this assumption limited our ability to examine reciprocity, which is a critical consideration in understanding the meaningful engagement and inclusion of civil society representatives in contributing to the application process. A deeper exploration of the nature of participation of key affected populations and civil society representatives within the Global Fund application network, including an examination of power dynamics, is warranted through future research examining the types of collaborative ties and their directionality.

## Conclusion

Our results indicate the 2017 Global Fund application processes in the DRC and Uganda were inclusive in terms of representation and participation and that respondents perceived the application process to be country-owned and effective; however, data also indicate perceptions of lower efficiency and higher financial costs. As part of process improvement, the Global Fund should consider examining the tradeoffs of a highly inclusive application development process, including striking the right balance between achieving inclusive and meaningful participation, responsiveness to country needs, and improved efficiency.

## Additional Files

The additional files for this article can be found as follows:

10.5334/aogh.2961.s1 Supplemental File 1. Description of Global Fund’s differentiated funding request review types.

10.5334/aogh.2961.s2 Supplemental File 2. Survey tools: Uganda (English) and DRC (French).

10.5334/aogh.2961.s3 Supplemental File 3. Additional network figures.

## References

[1] Rae J. Global Fund Overview. Published 2019 https://www.theglobalfund.org/en/overview/. Accessed December 15, 2019.

[2] The Global Fund. Investing to End Epidemics: The Global Fund Strategy 2017–2022. 2017.

[3] Sands P. Putting Country Ownership into Practice: The Global Fund and Country Coordinating Mechanisms. Heal Syst Reform. 2019; 5(2): 100–103. DOI: 10.1080/23288604.2019.1589831 31194640

[4] The Global Fund. Operational Policy Note: Access to Funding, Grant-making and Approval. Operational Policy Manual, v2.21. Published 2017 https://www.theglobalfund.org/media/3266/core_operationalpolicy_manual_en.pdf. Accessed August 5, 2019.

[5] Hanefeld J. The Global Fund to Fight AIDS, Tuberculosis and Malaria: 10 years on. Int Clin Med. 2014; 14(1): 54–57. DOI: 10.7861/clinmedicine.14-1-54 PMC5873622 24532746

[6] Brinkerhoff JM. Global public policy, partnership, and the case of the World Commission on Dams. Public Adm Rev. 2002; 62(3): 324–336. DOI: 10.1111/1540-6210.00182

[7] Kamya C, Shearer J, Asiimwe G, et al. Evaluating Global Health Partnerships: A Case Study of a Gavi HPV Vaccine Application Process in Uganda. Int J Heal Policy Manag. 2016; 6(6): 327–338. DOI: 10.15171/ijhpm.2016.137 PMC5458794 28812825

[8] Buse K, Harmer AM. Seven habits of highly effective global public–private health partnerships: Practice and potential. Soc Sci Med. 2007; 64(2): 259–271. DOI: 10.1016/j.socscimed.2006.09.001 17055633

[9] Brinkerhoff JM. Assessing and improving partnership relationships and outcomes: A proposed framework. Eval Program Plann. 2002; 25(3): 215–231. DOI: 10.1016/S0149-7189(02)00017-4

[10] Brinkerhoff JM. Partnership for International Development: Rhetoric or Results? Boulder, CO: Lynne Rienner Publishers; 2002.

[11] Itad. Thematic Review of the Global Fund Country Level Technical Support Partnership Model. 2019 https://www.theglobalfund.org/media/8792/terg_partnershipmodelreview_paper_en.pdf?u=637066527920000000.

[12] The Global Fund. Country Coordinating Mechanism Policy Including Principles and Requirements. Published 2018 https://www.theglobalfund.org/media/7421/ccm_countrycoordinatingmechanism_policy_en.pdf. Accessed August 5, 2019.

[13] The Global Fund. The Global Fund’s New Funding Model. 2013 https://www.theglobalfund.org/media/1467/replenishment_2013newfundingmodel_report_en.pdf?u=63648680736000000. Accessed August 20, 2019.

[14] Shearer JC, Lavis J, Abelson J, Walt G, Dion M. Evidence-informed policymaking and policy innovation in a low-income country: Does policy network structure matter? Evid Policy. 2018; 14(3): 381–401. DOI: 10.1332/174426418X15330477583836

[15] Armstrong R, Campbell White A, Chinyamuchiko P, Chizimbi S, Hamm Rush S, Poku NK. Inclusive engagement for health and development or “political theatre”: Results from case studies examining mechanisms for country ownership in Global Fund processes in Malawi, Tanzania and Zimbabwe. Global Health. 2019; 15(1): 1–11. DOI: 10.1186/s12992-019-0475-9 31064386 PMC6505082

[16] UNDP. Global Human Development Indicators. http://hdr.undp.org/en/countries. Accessed April 20, 2020.

[17] The Global Fund. Funding Model: Allocation. 2017–2019 Allocations. Published 2016 https://www.theglobalfund.org/en/funding-model/before-applying/allocation/. Accessed July 25, 2019.

[18] The Global Fund. Catalytic Investments. Published 2017 https://www.theglobalfund.org/en/funding-model/before-applying/catalytic-investments/#matching-funds. Accessed August 5, 2019.

[19] UNAIDS. UNAIDS Data 2019. https://www.unaids.org/sites/default/files/media_asset/2019-UNAIDS-data_en.pdf.

[20] WHO. Global Tuberculosis Report 2019. https://apps.who.int/iris/bitstream/handle/10665/329368/9789241565714-eng.pdf?ua=1.

[21] WHO. Incidence Data by country Global Health Observatory data repository. Published 2020 https://apps.who.int/gho/data/view.main.MALARIAINCIDENCEv?lang=en. Accessed April 29, 2020.

[22] The Global Fund. Global Fund Data Explorer. Published 2019 https://data.theglobalfund.org/locations. Accessed July 25, 2019.

[23] The Global Fund. Audit Report: Grant Oversight in Focused Portfolios. 2018 https://www.theglobalfund.org/media/8086/oig_gf-oig-18-022_report_en.pdf?u=637153280560000000.

[24] The Global Fund. Operational Policy Note: Challenging Operating Environments. Operational Policy Manual, v2.21. Published 2017 https://www.theglobalfund.org/media/3266/core_operationalpolicy_manual_en.pdf. Accessed August 5, 2019.

[25] The Global Fund. Eligibility List 2017. Published 2016 https://www.theglobalfund.org/media/5601/core_eligiblecountries2017_list_en.pdf. Accessed August 5, 2019.

[26] The Global Fund. Technical Evaluation Reference Group: Prospective Country Evaluations. https://www.theglobalfund.org/en/technical-evaluation-reference-group/prospective-country-evaluations/. Accessed June 10, 2019.

[27] PATH Health Systems Innovation and Delivery Program. Global Fund Prospective Country Evaluation: Uncovering what’s working, what’s not working, and why. Published 2019 https://www.path.org/programs/health-systems-innovation-and-delivery/global-fund-prospective-country-evaluation/. Accessed September 3, 2019.

[28] SurveyGizmo. Published 2017 www.surveygizmo.com.

[29] Knoke D, Yang S. Social Network Analysis Thousand Oaks, CA: SAGE Publications; 2007.

[30] Zaheer A, McEvily B, Perrone V. Does Trust Matter? Exploring the Effects of Interorganizational and Interpersonal Trust on Performance. Organ Sci. 1998; 9(2): 141–159. DOI: 10.1287/orsc.9.2.141

[31] Provan KG, Milward HB. A Preliminary Theory of Interorganizational Network Effectiveness: A Comparative Study of Four Community Mental Health Systems. Adm Sci Q. 1995; 40(1): 1–33. DOI: 10.2307/2393698

[32] Yang S, Keller FB, Zheng L. Social Network Analysis: Methods and Examples Thousand Oaks, CA: SAGE Publications, Inc; 2016 https://us.sagepub.com/en-us/nam/social-network-analysis/book241848 DOI: 10.4135/9781071802847

[33] Handcock MS, Hunter DR, Butts CT, Goodreau SM, Morris M. statnet: Software tools for the Statistical Modeling of Network Data. Published online 2003. 10.18637/jss.v024.i01 PMC2447931 18618019

[34] Beylerian EN, Jenness KL, Morris M. statnetWeb: A Graphical User Interface for Network Modeling with “Statnet.” Published online 2014.

[35] Gale NK, Heath G, Cameron E, Rashid S, Redwood S. Using the framework method for the analysis of qualitative data in multi-disciplinary health research. BMC Med Res Methodol. 2013; 13(1): 1 DOI: 10.1186/1471-2288-13-117 24047204 PMC3848812

[36] PATH, Institute for Health Metrics and Evaluation. Global Fund Prospective Country Evaluation: Democratic Republic of the Congo 2017–2018 Annual Report. 2018 https://www.path.org/resources/global-fund-prospective-country-evaluation-democratic-republic-congo-2018-annual-report/.

[37] Infectious Diseases Research Collaboration, Institute for Health Metrics and Evaluation, PATH. Global Fund Prospective Country Evaluation: Uganda 2017–2018 Annual Report. 2018 https://www.path.org/resources/global-fund-prospective-country-evaluation-uganda-2018-annual-report/.

[38] Blanchet K, Shearer J. Network analysis: A tool for understanding social network behaviour of a system In: de Savigny D, Blanchet K, Adam T (eds.), Applied Systems Thinking for Health Systems Research: A Methodological Handbook. London: Open University Press; 2017: 115–133.

[39] Blanchet K, James P. How to do (or not to do)…a social network analysis in health systems research. Health Policy Plan. 2012; 27(5): 438–446. DOI: 10.1093/heapol/czr055 21840934

[40] Popelier L. A scoping review on the current and potential use of social network analysis for evaluation purposes. Evaluation. 2018; 24(3): 325–352. DOI: 10.1177/1356389018782219

[41] Wonodi CB, Privor-Dumm L, Aina M, et al. Using social network analysis to examine the decision-making process on new vaccine introduction in Nigeria. Health Policy Plan. 2012; 27(Suppl.2). DOI: 10.1093/heapol/czs037 22513730

[42] RinGs. Adopting a Gender Lens in Health Systems Policy: A Guide for Policy Makers. 2020 https://ringsgenderresearch.org/resources/adopting-a-gender-lens-in-health-systems-policy-a-guide/.

[43] UNDP. Checklist for Integrating Gender into the Processes and Mechanisms of the Global Fund to Fight AIDS, Tuberculosis and Malaria. 2016 https://www.ghdonline.org/uploads/UNDP_Gender_Checklist_for_Global_Fund_Grants_2015.pdf.

[44] Warren A, Cordon R, Told M, de Savigny D, Kickbusch I, Tanner M. The Global Fund’s paradigm of oversight, monitoring, and results in Mozambique. Global Health. 2017; 13(1): 1–14. DOI: 10.1186/s12992-017-0308-7 29233165 PMC5728058

[45] The Global Fund. Step up the Fight: Focus on Universal Health Coverage. 2019 https://www.theglobalfund.org/media/5913/publication_universalhealthcoverage_focuson_en.pdf.

[46] Thuilliez J, Barré-Sinoussi F, Dabis F, Moatti JP, Yazdanpanah Y. The Global Fund in the era of SDGs: Time to rethink? Lancet Public Heal. 2020; 5(1): e17 DOI: 10.1016/S2468-2667(19)30249-X 31910978

[47] The Global Fund. Country Coordinating Mechanism Evolution. https://www.theglobalfund.org/en/country-coordinating-mechanism/evolution.

[48] Glandon D, Mondal S, Okeyo I, et al. Methodological gaps and opportunities for studying multisectoral collaboration for health in low- and middle-income countries. Health Policy Plan. 2019; 34: II7–II17. DOI: 10.1093/heapol/czz116 31723973

