# Supplementary material for: Partnership and Participation—A Social Network Analysis of the 2017 Global Fund Application Process in the Democratic Republic of the Congo and Uganda
Source: Ann Glob Health. 2020 Nov 5;86(1):140. doi: 10.5334/aogh.2961 (PMC7646284; doi:10.5334/aogh.2961)
Supplement: Supplemental File 1. — Description of Global Fund’s differentiated funding request review types. [file agh-86-1-2961-s1.pdf]

Partnership and participation—a social network analysis of the 2017 Global Fund application process in the Democratic Republic of the Congo and Uganda

Supplemental File 1. Description of Global Fund's differentiated funding request review types

Review type	Description	Key changes	Applications
Program Continuation	Permits grant implementation for an additional three years given the same assumptions of the current grant if no material changes have occurred in the scope and scale of the strategic focus and technical soundness and potential for impact.	• No new funding request but a 'self-assessment' submitted<br>• Technical Review Panel validation, not full review<br>• Performance framework and budget submitted at grant-making<br>• Grant-making focuses on updating previously agreed grant documents	DRC: Malaria
Tailored Review	Aimed at better matching specific objectives and applicant type with a view to streamlining the process. Tailored to the specific context and/or applicant such as COEs with material change in a defined programmatic area.	• Tailored funding request exists<br>• Tailored Technical Review Panel review<br>• More flexibility with Principal Recipient assessments and audit requirements at grant-making stage	DRC: TB/HIV
Full Review	Most akin to the review process for prior 2014-16 grant cycle. Requires budget and performance framework at initial submission.	Limited change relative to past grant cycle for 2014-2016 allocation period.	Uganda: Malaria<br>Uganda: TB/HIV

Differentiated Application and Review Approaches: The Global Fund Operational Policy Manual states that "based on agreed differentiation triggers, the Grant Approvals Committee (GAC) will determine the most suitable type of funding request and corresponding review approach for each country component. This was communicated to the applicant in the allocation letters sent in December 2016 but could change based on in-country discussions or the Technical Review Panel's assessment of the applicant's response. The Global Fund determined DRC was eligible to submit for program continuation and tailored review in part due to the lengthy delays in implementing the grants from the prior allocation period (2014-2016), meaning not much change in strategy was anticipated in moving to the 2017-2019 allocation.
