# Supplementary material for: Partnership and Participation—A Social Network Analysis of the 2017 Global Fund Application Process in the Democratic Republic of the Congo and Uganda
Source: Ann Glob Health. 2020 Nov 5;86(1):140. doi: 10.5334/aogh.2961 (PMC7646284; doi:10.5334/aogh.2961)
Supplement: Supplemental File 2. — Survey tools: Uganda (English) and DRC (French). [file agh-86-1-2961-s2.pdf]

Supplemental File 2 – Survey tools: Uganda (English) and DRC (French)

A. Uganda Partnership Network Survey / Prospective Country Evaluation

---

Purpose of the Study
The purpose of the **Global Fund Prospective Country Evaluation** study is to gather information about the activities related to the implementation of Global Fund investments in Uganda. This survey will ask questions about the nature of your collaboration with other individuals and organizations.

Who is organizing the Study?
The study is being organized Infectious Diseases Research Collaboration to understand the role and function of partnerships between the Global Fund, partners, and in-country stakeholders in carrying out the activities in the grant application and implementation processes in Uganda.

Confidentiality:
Your responses will be made anonymous and all data will be kept confidential.
This means your name will not be used in any presentation of network results; all data will be aggregated and summarized at the organizational level.

---

1) Do you agree to participate in this survey?

☐ Yes

☐ No → SURVEY END

About yourself

2) What is your name? (First Last)

Type first-name last-name. Most names will auto-complete when you begin typing the first name. If they do not, please go ahead and type the name (e.g., Grace Chan)

____

3) Please select your gender:

☐ Male

☐ Female

☐ Prefer not to answer

☐ Other, write in: ______________________________

4) What organization, and division within the organization, did you work for (or represent) during the 2017-2019 Global Fund application cycle?

Note to interviewer: *emphasize that here we are looking for information about the organization that pays the person's salary (not CCM affiliation, for example). In Q5, you can confirm if they are also part of the CCM or have affiliation with any other organization.*

Organization: ____

Division/Department/Program: ____

5) What type of organization(s) do you work?: (select all that apply; be sure to note whether the person is on the CCM and/or if they are a PR or SR).

( ) Government – Ministry of Health/Malaria program

( ) Government – Ministry of Health/HIV/AIDS program

( ) Government – Ministry of Health/TB program

( ) Government – Ministry of Health/Health Management Information System (HMIS) unit

( ) Government – Ministry of Health/Other, Specify __________________________

( ) Government – Ministry of Finance

( ) Government – Other, Specify ___________________________

( ) Global Fund Principal Recipient

( ) Global Fund Sub-Recipient

( ) Development partner (e.g. WHO, UNAIDS, STOP TB Partnership, Roll Back Malaria, etc.)

( ) Country Coordinating Mechanism

( ) The Global Fund (e.g. LFA, fiscal agent, Country Team)

( ) Researcher/academic

( ) Consultant

( ) Civil society

( ) NGO

( ) Other funder/donor, Specify ________________________________

6) Please indicate which Global Fund application component you participated in the 2017-19 cycle? Select all that apply.

[ ] HIV/AIDS

[ ] TB

[ ] Malaria

For each application that you worked on, please indicate which parts of the process and/or application components you worked on.

7.) For the (Malaria/ HIV/TB) application, please indicate ("X") which parts of the process and/or application components you were involved

**Process steps:**	**HIV/AIDS**	**TB**	**Malaria**	**N/A**
Preparatory activities leading up to the grant application<br><br>***Note:*** *These could include evidence and data reviews, reviewing past performance, changes in epidemiological situation, gap analyses, impact and resource allocation modeling.*				
Developing the application for funding<br><br>***Note:*** *The application for funding is submitted to Global Fund by the CCM (this was previously called "concept note"*)				
Grant making process and negotiations<br><br>***Note:*** *Grant making is the process beginning after the application is approved by the TRP. It is the process of negotiating the grant agreement with Global Fund.*				

Providing technical assistance<br><br>***Note:*** *TA involves engagement to strengthen the capacity of individuals and institutions to undertake relevant activities, such as funding request development.*				
Health systems strengthening / RSSH				
Key and vulnerable populations considerations				
Gender considerations				
Co-financing (including negotiations with the government to increase domestic spending in health)				
Sustainability strategy (including: the Funding Landscape Table and the Financial Gap Worksheet)				
Performance framework / M&E plan				
Budget				

Collaboration (Q # 8-10 in the online survey)

8) We would now like to ask you about who you collaborated or worked together with during the 2017 funding request and/or grant making process (across all applications that you worked on). For example, by working together you may have exchanged information to support the funding request, cooperated on writing sections of the funding request, collaborated on setting performance targets and developing action plans, or responded to reviewer comments, etc.)

Please provide the name (first and last) of the individuals you worked with. They can be within or outside your own organization. *Most names will auto-complete when you begin typing the first name. If they do not, please go ahead and type the name (e.g., Grace Chan). You can enter as many as* ***ten names****.*

9) For each of the names you provided, please indicate which application component you worked on together (you may select multiple components).

10) Now I would like you to reflect on the level of trust you have for the individual and/or their organization. Can you describe your trust for that person/organization that you collaborate with. When we say trust, we mean you can trust that individual or organization to keep their work, do a good job, and respond to your professional needs or your organization's

needs. The trust scale is from 1 to 4, as follows: 1 = Poor relationship (Little Trust); 2 = Fair relationship (Some Trust); 3 = Good relationship (Trust); 4 = Excellent relationship (High Trust); No response

**Q8: Name and Organization of individuals worked with on the 2017-19 application cycle** (up to 10)		**Q 9: Type of collaboration (by grant component)**			**Q10: Trust level**
**Name**	**Organization**	**HIV/AIDS**	**TB**	**Malaria**	**Circle one response**
1.		[ ]	[ ]	[ ]	1 2 3 4 / No response
2.		[ ]	[ ]	[ ]	1 2 3 4 / No response
3.		[ ]	[ ]	[ ]	1 2 3 4 / No response
4.		[ ]	[ ]	[ ]	1 2 3 4 / No response
5.		[ ]	[ ]	[ ]	1 2 3 4 / No response
6.		[ ]	[ ]	[ ]	1 2 3 4 / No response
7.		[ ]	[ ]	[ ]	1 2 3 4 / No response
8.		[ ]	[ ]	[ ]	1 2 3 4 / No response
9.		[ ]	[ ]	[ ]	1 2 3 4 / No response
10.		[ ]	[ ]	[ ]	1 2 3 4 / No response

Who was most influential?

For each funding request that you worked on, we would now like to discuss which individuals were the most influential. For example, by "influential", we mean the person was instrumental in decision-making, had a strong voice, exerted power in steering the conversation and in directing the strategic focus of the funding request and/or grant making process.

11 and 12) For the TB/HIV and malaria funding requesst, considering all individuals who participated in the application process (even if you did not work together directly), please name the top three most influential individuals.

Type first-name last-name. Most names will auto-complete when you begin typing the first name. If they do not, please go ahead and type the name (e.g., Grace Chan). You can enter as many as three names.

	**Q11: TB/HIV funding request**	**Q12: Malaria funding request**
**Name 1**<br>**Organization 1**		
**Name 2**<br>**Organization 2**		
**Name 3**<br>**Organization 3**		

Partnership added-value scorecard

Our last question has to do with understanding what are the benefits and drawbacks of working together in partnership with other individuals and organizations during the 2017-19 Global Fund application.

13) For each possible benefit listed, please indicate, by placing a check in the appropriate box, whether you think that because of working together with other individuals and organizations, the benefit occurred or not. Check (☐) only one box for each benefit.

**Benefits:**	**Occurred**	**Did not occur**	**Don’t know / NA**
a. Better able to execute activities	☐	☐	☐
b. More timely execution of planned activities	☐	☐	☐
c. Increased quality / technical soundness of the approved grants	☐	☐	☐
d. Better able to identify the need for, and to acquire additional technical support	☐	☐	☐

e. Better able to respond to challenges and bottlenecks that arose during funding request/grant making process	☐	☐	☐
f. Increased inclusiveness of key stakeholders in the process (e.g. key populations, community organizations, etc.)	☐	☐	☐
g. Reduction in financial cost of process	☐	☐	☐
h. Leveraged each organizations' comparative advantages	☐	☐	☐
i. Reduced transaction costs (i.e. more streamlined grant application process)	☐	☐	☐
j. Approved grants that are more responsive to country needs	☐	☐	☐
k. Increased transparency among partners	☐	☐	☐
l. Increased accountability among partners	☐	☐	☐
m. Increased legitimacy of decisions made	☐	☐	☐
n. Increased fairness of decisions made	☐	☐	☐

14) Other benefits, specify: ____

15) For each possible drawback listed, please indicate, by placing a check in the appropriate box, whether you think that because of working together with other individuals and organizations, the drawback occurred or not. Check (☐) only one box for each drawback.

**Drawbacks:**	**Occurred**	**Did not occur**	**Don't know / NA**
a. Unnecessary management burden on my organization	☐	☐	☐
b. Created competition and conflict among member organizations	☐	☐	☐
c. Loss of control/autonomy over decisions	☐	☐	☐
d. Strained relations within my organization	☐	☐	☐
e. Not enough credit given to my organization	☐	☐	☐
f. Forced us to make decisions in a way which was not natural/typical for our organization	☐	☐	☐

16) Other drawbacks, specify: ____

17) If we have any questions regarding your survey would you be able to provide the best email or phone number to contact you?

Email: ___________________________

Phone: ___________________________

Survey End

Thank You!

A. DRC Evaluation Prospective du Pays / Fond mondial

Enquête sur le réseau de partenariat

____

A propos de vous-même

1) Comment vous appelez-vous? (Prénom Nom)

Tapez le nom du interviewe. La plupart des noms se compléteront lorsque vous commencez à taper le prénom. S'ils ne le font pas, veuillez aller de l'avant et tapez le nom (p.e. Grace Chan).

____

2) Veuillez choisir votre sexe: ☐ Homme ☐ Femme

3) Veuillez taper votre adresse email :

____

4) Pour quelle organisation vous avez travaillé (ou quelle organisation vous avez représenté) lors du processus de demande de financement du cycle 2017-2019? Et dans quelle division, unité, ou programme vous avez travaillé ? __________________________________

5) à remplir par l'enquêteur : cocher la case qui s'applique au type d'organisation: (sélectionnez tout ce qui s'applique ; assurez-vous de noter si la personne appartient au CCM et/ou si elle est PR ou SR).
( ) MSP – PNLS
( ) MSP – PNLP
( ) MSP – PNLT
( ) MSP – SNIS
( ) Ministère du genre, famille et de l'enfant
( ) Ministère de la justice et des droits humains
( ) Ministère de Finance
( ) Gouvernement – Autre
( ) Société civile – ONG locale
( ) Société civile – ONG internationale
( ) Récipiendaire principal du FM
( ) Sous-Récipiendaire du FM

( ) Partenaire de développement (p.e. OMS, ONUSIDA, STOP TB Partnership, Roll Back Malaria, etc.)
( ) Mécanisme de coordination du pays (CCM)
( ) Le Fonds mondial (p.e. LFA, agent fiscal, Equipe du pays)
( ) Autre bailleur de fonds/donateur
( ) Chercheur/académique

6) Veuillez indiquer à quels processus de demande de financement du FM vous avez participé au cours du cycle 2017-19 ? Choisissez tout thématique ce qui s'applique.
[ ] VIH/SIDA
[ ] Tuberculose
[ ] Malaria

Pensez à la demande de financement de 2017 du FM. Pour chaque demande de financement sur laquelle vous avez travaillez, nous voudrions savoir dans quelles étapes du processus et dans quelles thématiques vous avez participé.

7) Pour la demande de financement de (VIH/TB ou paludisme), veuillez indiquer dans quelles étapes du processus et dans quelles thématiques vous avez participé.

**Etapes du processus:**	
[ ] J'ai collabore sur les activités préparatoires qui ont eu lieu avant le développement de la demande de financement	***Note:*** *Ça pourrait inclure revues de programme, revues de l'évidence, revues sur les données épidémiologiques et sur la situation, analyses de gap, analyses sur l'impact et l'allocation de ressources.*
[ ] J'ai collabore sur le développement de la demande de financement	***Note:*** *La demande de financement est soumise par le CCM au FM*
[ ] J'ai collaboré sur le développement de l'accord de fonds (l'octroi) avec le FM, compris les négociations	***Note:*** *Le développement de l'accord de fonds est le processus commençant après l'approbation de la demande de financement par le TRP, y compris les négociations entre PR et FM*
[ ] J'ai fourni une assistance technique	***Note:*** *L'AT implique l'engagement pour renforcer la capacité des individus et des institutions et fournir un appui à la rédaction de la demande de financement.*

[ ] Renforcement du système de sante / RSSH

[ ] Considérations par rapport aux populations clés et vulnérables

[ ] Considérations par rapport au genre

[ ] Co-financement (inclus les négociations avec le gouvernement afin d'accroitre les dépenses nationaux sur la sante)

[ ] Stratégies visant la durabilité (inclut: the Funding Landscape Table and the Financial Gap Worksheet)

[ ] Cadre de performance / Plan de S&E

[ ] Budget

____

Collaboration

Maintenant nous allons passer sur quelques questions afin de comprendre quels sont les individus avec qui vous avez collaboré ou travaillé lors de processus de demande de financement de 2017.

7) Prier d'identifier avec qui vous avez collaboré ou travaillé le plus souvent (compris tous les différentes demandes de financement si vous en avez travaillez sur plusieurs). Vous pouvez indiquer jusqu'à dix personnes.

En travaillant ensemble, ça veut dire que vous avez, par exemple, échangé des informations pour soutenir la requête de financement, que vous avez collaboré sur la rédaction de la demande de financement, sur la détermination des cibles, sur le développement des plans d'actions, ou sur les répondre aux commentaires du TRP, etc…)

Tapez le prénom – le nom et l'organisation de la personne. La plupart des noms s'auto compléteront lorsque vous commencez à taper le prénom. S'ils ne le font pas, veuillez aller de l'avant et tapez le nom (p.ex. Grace Chan). Veuillez indiquer avec quelle organisation le personne travail

Nom 1: _______________________________ Organisation: _______________________________

Nom 2: _______________________________ Organisation: _______________________________

Nom 3: _______________________________ Organisation: _______________________________

Nom 4: _______________________________ Organisation: _______________________________

Nom 5: _______________________________ Organisation: _______________________________

Nom 6: _______________________________ Organisation: _______________________________

Nom 7: _______________________________ Organisation: _______________________________

Nom 8: _______________________________ Organisation: _______________________________

Nom 9: _______________________________ Organisation: _______________________________

Nom 10: _______________________________ Organisation: _______________________________

8) Pour chacun des noms que vous avez donnés, veuillez indiquer sur quelle component de la requête de financement vous avez collaboré pour le cycle 2017-19 (vous pouvez sélectionner plusieurs components).

	**Components de la requête de financement**		
**Nom**	**VIH/SIDA**	**Tuberculose**	**Malaria**
[question("valeur"), id="collab1"]	[ ]	[ ]	[ ]
[question("valeur"), id="collab2"]	[ ]	[ ]	[ ]
[question("valeur"), id="collab3"]	[ ]	[ ]	[ ]
[question("valeur"), id="collab4"]	[ ]	[ ]	[ ]
[question("valeur"), id="collab5"]	[ ]	[ ]	[ ]
[question("valeur"), id="collab6"]	[ ]	[ ]	[ ]
[question("valeur"), id="collab7"]	[ ]	[ ]	[ ]
[question("valeur"), id="collab8"]	[ ]	[ ]	[ ]
[question("valeur"), id="collab9"]	[ ]	[ ]	[ ]
[question("valeur"), id="collab10"]	[ ]	[ ]	[ ]

10) Maintenant, j'aimerais que vous puissiez nous refléter le niveau de confiance que vous avez envers la personne et/ou leur organisation. Quand nous disons confiance, nous voulons dire que vous pouvez compter sur cette personne ou cette organisation pour garder sa parole, faire un bon travail, et/ou satisfaire vos besoins professionnels ou les besoins de votre organisation.

	**Niveau de confiance**				
	**1 Mauvaise relation (Peu de confiance)**	**2 Relation passable (Un peu de confiance)**	**3 Bonne relation (Confiance)**	**4 Excellente relation (Grande confiance)**	**Sans réponse**
[question("valeur"), id="collab1"]	[ ]	[ ]	[ ]	[ ]	[ ]
[question("valeur"), id="collab2"]	[ ]	[ ]	[ ]	[ ]	[ ]
[question("valeur"), id="collab3"]	[ ]	[ ]	[ ]	[ ]	[ ]
[question("valeur"), id="collab4"]	[ ]	[ ]	[ ]	[ ]	[ ]
[question("valeur"), id="collab5"]	[ ]	[ ]	[ ]	[ ]	[ ]

[question("valeur"), id="collab6"]	[ ]	[ ]	[ ]	[ ]	[ ]
[question("valeur"), id="collab7"]	[ ]	[ ]	[ ]	[ ]	[ ]
[question("valeur"), id="collab8"]	[ ]	[ ]	[ ]	[ ]	[ ]
[question("valeur"), id="collab9"]	[ ]	[ ]	[ ]	[ ]	[ ]
[question("valeur"), id="collab10"]	[ ]	[ ]	[ ]	[ ]	[ ]

Qui était le plus influent?

Maintenant, pour chaque demande de financement sur lequel vous avez travaillé, nous voudrions savoir quels sont les personnes les plus influentes. Par exemple, par 'influent' nous voulons dire que la personne a joué un rôle clé ou déterminant dans les processus décisionnels, avait une voix importante, a exercé le pouvoir en dirigeant la conversation et en fournissant l'orientation stratégique de la demande de financement et / ou du processus de développement de la subvention.

11) Pour le requête de financement VIH/TB ou paludisme, prenant en considération toutes les personnes qui ont participé au processus (even if you did not work together directly), veuillez nommer les trois personnes les plus influentes.
Tapez le prénom – le nom. La plupart des noms s'auto compléteront quand vous commencez à taper le prénom. S'ils ne le font pas, veuillez aller de l'avant et taper le nom (p.ex. Grace Chan).

	**Nom**	**Organisation**
Nom 1		
Nom 2		
Nom 3		

____

La carte des scores de la valeur ajoutée des partenariats

12) Maintenant, nous voudrions savoir quels étaient les avantages et les inconvénients du travail en commun et de la collaboration en partenariat avec d'autres personnes et organisations dans le contexte de la demande 2017-19 du FM. Pour chaque avantage ou inconvénient possible inscrite sur la liste, veuillez indiquer, si vous pensez que parce que vous avez travaillé ensemble avec d'autres personnes ou organisations, l'avantage ou l'inconvénient liste est arrivé ou non.

Marquez seulement dans une case pour chaque avantage /inconvénient.

**Avantages**	**Est arrivé**	**N'est pas arrivé**	**Ne sait pas**
o. Plus capable d'exécuter des activités	☐	☐	☐
p. Plus d'exécution à temps des activités planifiées	☐	☐	☐
q. Amélioration de la qualité / solidité technique des subventions approuvées	☐	☐	☐
r. Plus capable d'identifier le besoin pour, et d'acquérir l'appui technique additionnel	☐	☐	☐
s. Plus capable de faire face aux défis et goulots d'étranglements identifiés dans la requête de financement ou le processus de développement de la subvention.	☐	☐	☐
t. Accroitre l'inclusion des parties prenantes-clés dans le processus (p.ex. les populations-clés, organisations communautaires, etc)	☐	☐	☐
u. Réduction du coût financier du processus	☐	☐	☐
v. La favorisation des avantages comparatifs de chaque organisation	☐	☐	☐
w. La réduction des coûts des transactions (c.à.d. le processus de demande de fonds plus alignée)	☐	☐	☐
x. Les fonds approuvés qui répondent plus aux besoins du pays	☐	☐	☐
y. Plus de transparence parmi les partenaires	☐	☐	☐
z. Responsabilité accrue parmi les partenaires	☐	☐	☐
aa. Plus de légitimité dans la prise de décision	☐	☐	☐
bb. Plus de justesse dans les décisions prises	☐	☐	☐
cc. Autres bénéfices (à préciser) ____			

**Inconvénients:**	**Est arrivé**	**N’est pas arrivé**	**Ne sait pas**
a. Un fardeau superflu de gestion sur mon organisation	☐	☐	☐
b. Concurrence et conflit créés entre les staffs des organisations	☐	☐	☐
c. Perte de contrôle/d’autonomie sur les décisions	☐	☐	☐
d. Relations tendues au sein de mon organisation	☐	☐	☐
e. Pas assez de confiance accordée à mon organisation	☐	☐	☐
f. Nous a forcés à prendre des décisions d’une manière qui n’était pas naturelle/typique pour notre organisation	☐	☐	☐
g. Autres inconvénient (à préciser): ______________________________			

Merci!
