# Supplementary material for: Partnership and Participation—A Social Network Analysis of the 2017 Global Fund Application Process in the Democratic Republic of the Congo and Uganda
Source: Ann Glob Health. 2020 Nov 5;86(1):140. doi: 10.5334/aogh.2961 (PMC7646284; doi:10.5334/aogh.2961)
Supplement: Supplemental File 3. — Additional network figures. [file agh-86-1-2961-s3.pdf]

Partnership and participation—a social network analysis of the 2017 Global Fund application process in the Democratic Republic of the Congo and Uganda

Supplemental File 3. Additional network figures

Figure 1. Application networks by Funding Request type, plotted by gender.

a. Uganda: TB/HIV (Full Review)

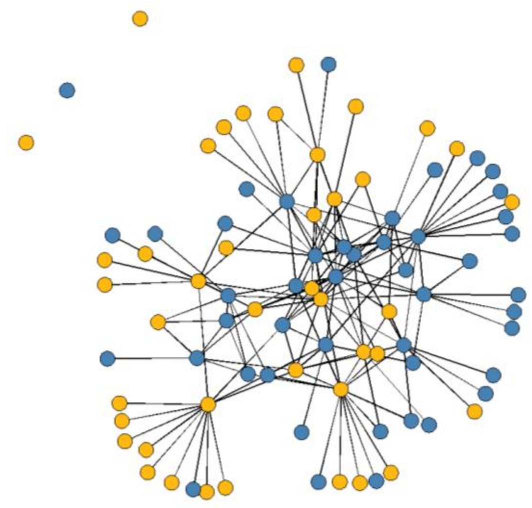

b. DRC: TB/HIV (Tailored Review)

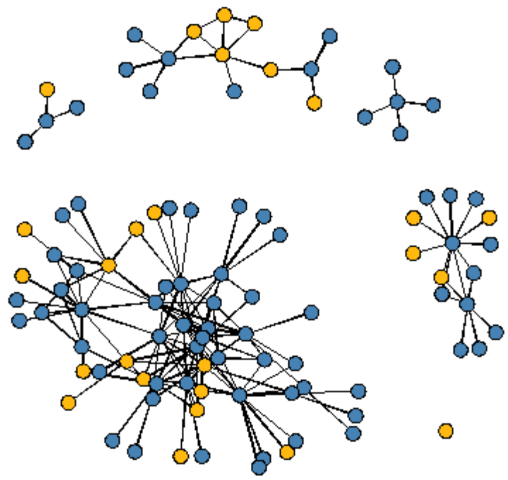

c. Uganda: Malaria (Full Review)

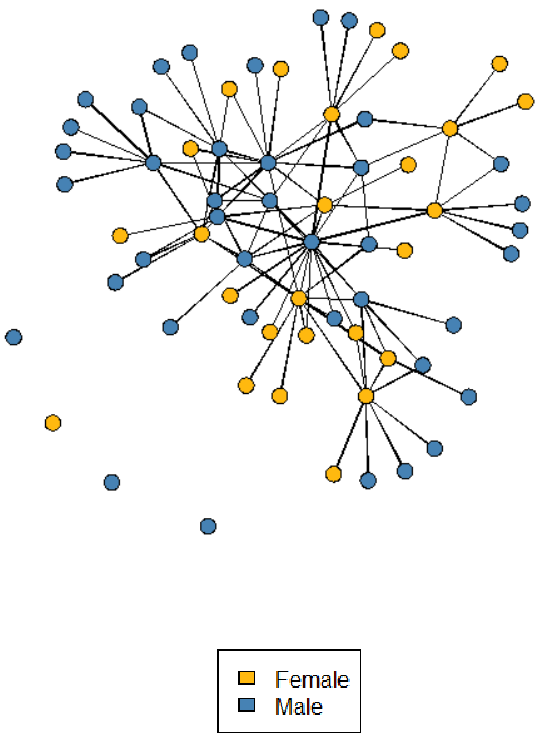

d. DRC: Malaria (Program Continuation)

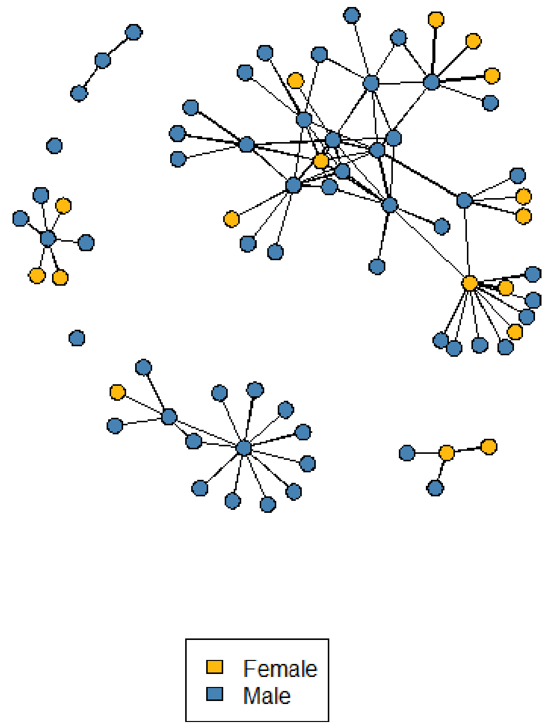

Figure 2. Plots of application networks with nodes sized according to betweenness centrality scores.

Plots of Uganda's 2017 Global Fund application networks with nodes sized by betweenness centrality scores and color coded to represent funding request type, organizational affiliation, and gender.

UNDERLINE_PLACEHOLDER

Funding Request

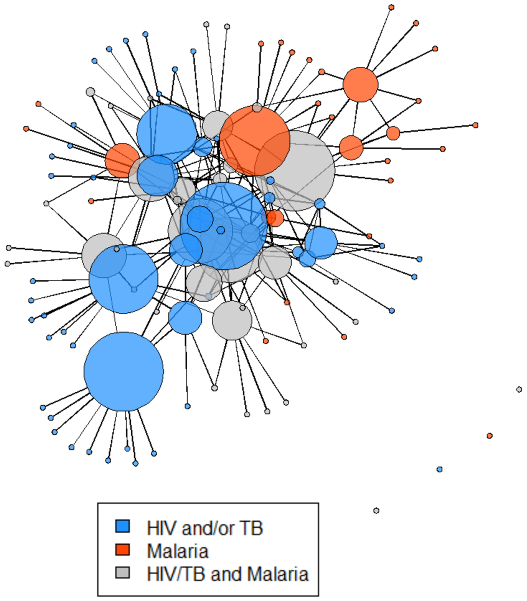

Organizational Affiliation

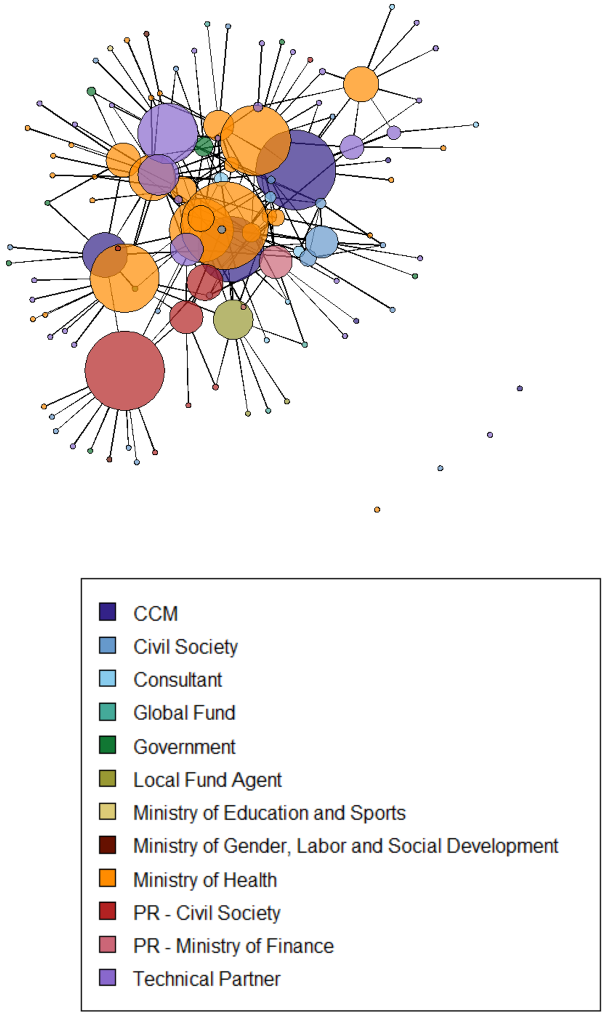

Gender

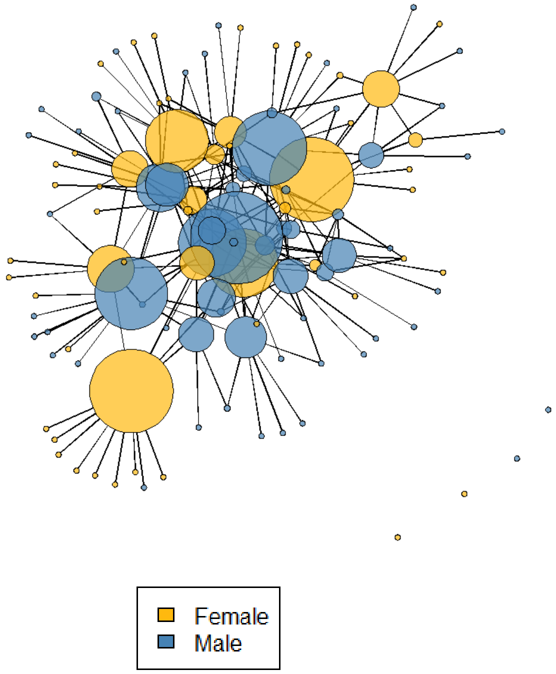

Partnership and participation—a social network analysis of the 2017 Global Fund application process in the Democratic Republic of the Congo and Uganda

Plots of DRC's 2017 Global Fund application networks with nodes sized by betweenness centrality scores and color coded to represent funding request type, organizational affiliation, and gender.

DRC

Funding Request

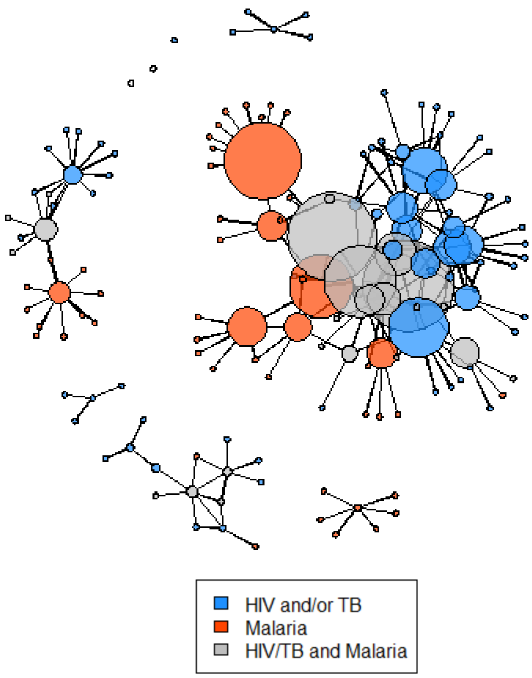

Organizational Affiliation

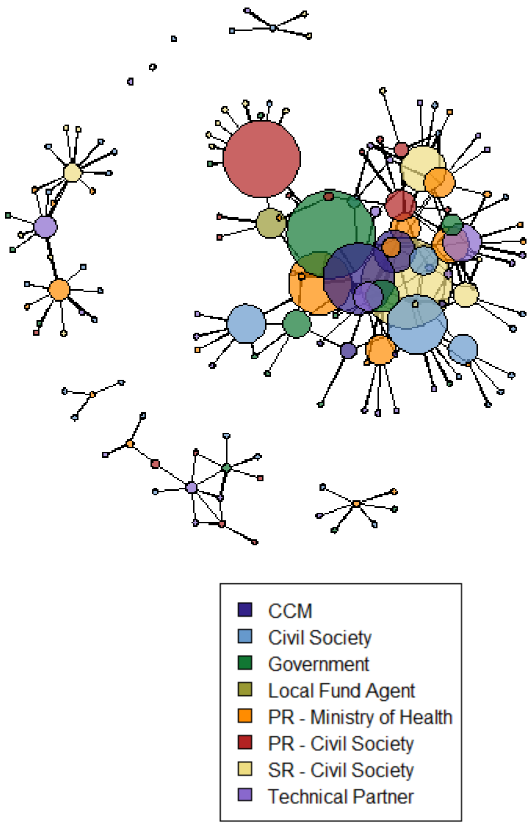

Gender

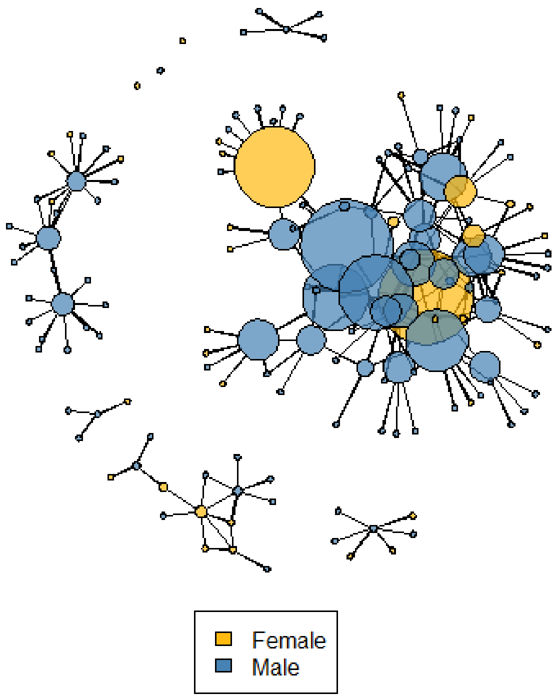

Figure 3. Application networks by Funding Request type, plotted by organizational affiliation.

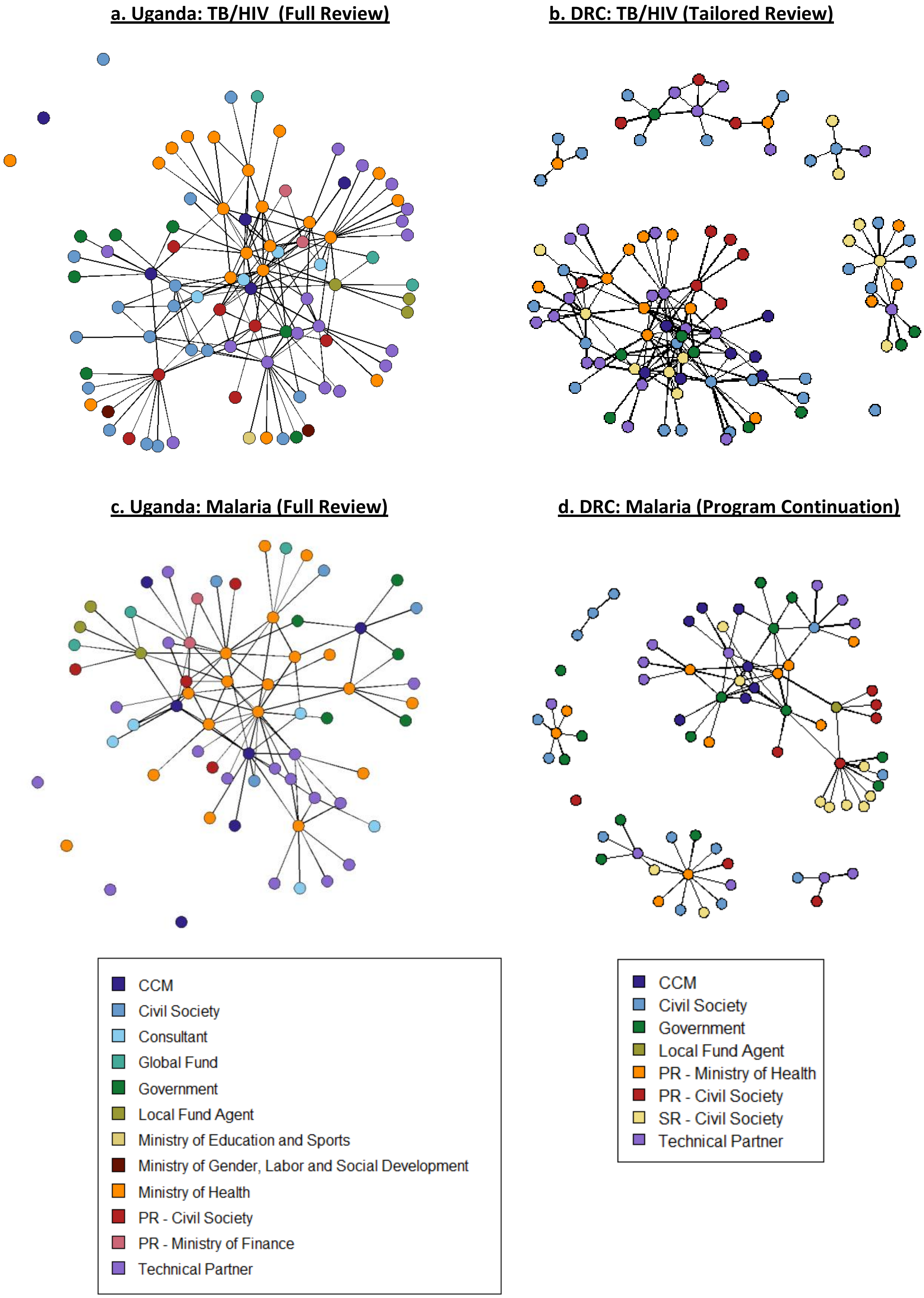

Partnership and participation—a social network analysis of the 2017 Global Fund application process in the Democratic Republic of the Congo and Uganda
